# Endothelial Nitric Oxide Synthase Mediates the Cerebrovascular Effects of Erythropoietin in Traumatic Brain Injury

**DOI:** 10.3389/fimmu.2014.00494

**Published:** 2014-10-09

**Authors:** Jovany Cruz Navarro, Shibu Pillai, Lucido L. Ponce, Mai Van, Jerry Clay Goodman, Claudia S. Robertson

**Affiliations:** ^1^Department of Neurosurgery, Baylor College of Medicine, Houston, TX, USA; ^2^Department of Pathology and Immunology, Baylor College of Medicine, Houston, TX, USA; ^3^Department of Neurology, Baylor College of Medicine, Houston, TX, USA

**Keywords:** erythropoietin, traumatic brain injury, nitric oxide, nitric oxide synthase, neuroprotection, cerebral blood flow

## Abstract

**Background:** Erythropoietin (Epo) improves post-traumatic cerebral blood flow (CBF), pressure autoregulation, and vascular reactivity to l-arginine. This study examines the dependence of these cerebral hemodynamic effects of Epo on nitric oxide generated by endothelial nitric oxide synthase (eNOS).

**Methods:** Using laser Doppler flow imaging, CBF was monitored in wild-type (WT) and eNOS-deficient mice undergoing controlled cortical impact followed by administration of Epo (5000 U/kg) or normal saline.

**Results:** Cerebral blood flow decreased in all groups post-injury with the greatest reductions occurring at the impact site. Epo administration resulted in significantly higher CBF in the peri-contusional sites in the WT mice [70.2 ± 3.35% in Epo-treated compared to 53 ± 3.3% of baseline in saline-treated mice (*p* < 0.0001)], but no effect was seen in the eNOS-deficient mice. No CBF differences were found at the core impact site where CBF dropped to 20–25% of baseline in all groups.

**Conclusion:** These differences between eNOS-deficient and WT mice indicate that the Epo mediated improvement in CBF in traumatic brain injury is eNOS dependent.

## Introduction

Traumatic brain injury (TBI) remains a major public health problem in the United States. Each year, an estimated 1.7 million people sustain some kind of TBI (1). More than 50,000 people die, and a larger number of head injured patients suffer permanent disability. Despite many promising experimental neuroprotection experiments, there is presently no effective treatment that promotes functional recovery in clinical TBI (2).

A substantial body of evidence has shown that erythropoietin (Epo) has extra-erythropoietic activities including neural protective effects against a wide variety of acute experimental insults, including spinal cord ischemia and trauma (3, 4), cerebral ischemia (5), bilateral carotid occlusion (6), retinal ischemia caused by uncontrolled intracranial pressure (7), subarachnoid hemorrhage (8), and subdural hematoma (9). Administration of Epo and Epo-analogs in experimental models of TBI leads to functional, morphological, and cognitive recovery that has been attributed to a several cytoprotective mechanisms including inhibition of apoptosis, anti-oxidant and anti-inflammatory actions, improved tissue oxygenation due to vascular effects, and stimulation of neurogenesis and angiogenesis (10–21). In the present study, we examined the effect of Epo on cerebral blood flow (CBF) and the dependence of this effect on endothelial nitric oxide synthase (eNOS) following controlled cortical impact (CCI).

### Background

Erythropoietin is a 34-kDa glycoprotein consisting of 165 amino acids residues, synthesized mainly by the fetal liver and adult kidney that is responsible for the proliferation, survival, and differentiation of erythroid progenitor cells (22, 23). Epo and erythropoietin receptor (EpoR) expression in the neonatal and adult brain are up-regulated during tissue hypoxia through an oxygen-sensing pathway mediated by hypoxia-inducible factors (24, 25). Epo acts by binding to a transmembrane receptor (EpoR) that belongs to the type I cytokine family, causing dimerization of the receptor, activating Janus-tyrosine-kinase-2 (JAK-2) that leads to several downstream signaling pathways involved in the hematopoietic and extra-hematopoietic effects of Epo (23, 26, 27).

The mechanism of Epo neuroprotection is not entirely clear, and several pathways may be involved, including cerebrovascular effects through alterations in nitric oxide (NO) production by eNOS. In ischemia models, this neuroprotection may be mediated by hypoxia-inducible factor 1 (HIF-1) activating its target genes Epo, EpoR, and vascular endothelial growth factor (VEGF) (28–30). Alternatively, it has been suggested that Epo acts indirectly on endothelial cells via activation of the VEGF receptor system, which is the most important specific regulator of endothelial growth and a major angiogenesis factor. *In vitro* studies, suggest that Epo is a promising candidate in the treatment of cerebral aneurysms by increasing the expression of eNOS and VEGF (31). Epo administration to coronary artery endothelial cells up-regulates eNOS activity leading to enhance NO production (32). In an isolated rat heart ischemia-reperfusion model, pretreatment with Epo provides cardioprotection that is dependent on NO (33). NO also has an important neuroprotectant role in TBI. In rodent models of TBI, administration of l-arginine increases NO levels in cerebral microdialyzate, restores CBF to near pre-injury levels and reduces the resulting contusion volume (34). Pre-injury administration of Epo in an experimental TBI model in rats treated with l-arginine results in a significant CBF recovery, likely through a mechanism involving eNOS activity (18). In experimental intracerebral hemorrhage, Epo reduces inflammation around the hematoma and activates eNOS possibly leading to improved perfusion (35). When diffuse axonal injury is coupled with hypoxia, Epo administration results in improved motor and cognitive performance, improved neuritic sprouting, and less corpus callosum thinning (36).

The purpose of this study was to evaluate the role of eNOS in the vascular effects of Epo after CCI in wild type (WT) compared to eNOS knock-out mice. Our hypothesis was that Epo mediated effects on cerebrovascular physiology are eNOS dependent.

## Materials and Methods

This protocol was approved by the institutional animal protocol review committee, using guidelines for humane care and use of animals that were developed for the National Institutes of Health (NIH).

### Cerebral blood flow experiments

A total of 56 adult male WT eNOS+/+ mice (C57BL/6J, *n* = 28) and homozygous eNOS-deficient mice (B6129P2-Nos tm I Unc/J with a C57BL/6 genetic background, *n* = 28; Jackson Laboratories, Bar Harbor, ME) were used to study the CBF response after Epo administration during the first 2 h of monitoring after experimental TBI. eNOS-deficient animals used in this experiment were the offspring of mating between heterozygous eNOS-deficient mice. The eNOS-deficient mice were produced using 1.2 kb neomycin cassette inserted into eNOS replacing 129 base pairs of exon 12 of the gene, disrupting the calmodulin binding site of the eNOS and introducing a premature stop codon in the transcripts. Disruption of the eNOS gene was confirmed by PCR. eNOS protein in cerebral cortex determined by western blotting was 40% that seen in WT animals.

eNOS-deficient and WT mice (14 mice per group) were randomly treated with Epo (5000 U/kg) or 0.5 ml of normal saline (NS) administered intra-peritoneally (IP) 5 min after the controlled cortical injury. The different experimental groups included the following:

eNOS+/−[NS] = eNOS-deficient mice treated with NS
eNOS+/−[Epo] = eNOS-deficient mice treated with Epo
WT-[NS] = Wild-type mice treated with NS
WT-[Epo] = Wild-type mice treated with Epo

The mice were anesthetized with 5% isoflurane in 100% oxygen in a vented anesthesia chamber and maintained on 2% isoflurane for the duration of the experiment using a volume-controlled ventilator (Hugo Sachs Elektronik-Harvard Apparatus, March-Hugstetten, Germany) adjusted to obtain and end-tidal CO2 level [EtCO2] between 35 and 40 mm Hg monitored with microcapnography (Columbus Instruments, Columbus, OH, USA). Rectal temperature was maintained between 36 and 37°C using a heat pad and a rectal thermistor. Blood pressure was monitored via femoral artery cannulation.

Baseline mean arterial pressure (MAP) and CBF were obtained 15 min prior to the cortical impact and thereafter, MAP was monitored at 30-s intervals. CBF was measured immediately after Epo or NS injection at 5 min after CCI and then at 30, 60, 90, and 120 min post-injury using a non-invasive laser Doppler flow (LDF) scanner (PIM3 System, Perimed AB Stockholm, Sweden). Periscan PIM3 measures the total microcirculatory blood perfusion including the perfusion in capillaries, arterioles, venules, and shunting vessels. Measurements from this instrument are expressed as arbitrary perfusion units (PU). To enable comparison of results, perfusion changes were expressed as percentage of baseline measurements. The LDF device was positioned 15 cm above the head, and PUs were measured in regions of interest (ROIs) ipsi- and contra-lateral to the injury. The ROIs consisted of the site of the impact, cerebral cortex adjacent to the impact site (peri-contusional), and the contra-lateral cerebral hemisphere.

Controlled cortical impact injury was produced as previously described (34). In brief, after craniectomy and dural exposure, the 3 mm diameter impactor tip was positioned perpendicular to the exposed surface of the brain at an angle of 45° to the vertical. CCI (3 m/s, 1.5 mm deformation, 100 ms of duration) was performed using a voltage driven impactor (Benchmark Stereotaxic Impactor, myNeuroLab, St Louis, MO, USA).

### Statistical analysis

Outcome assessments that were repeated over time such as CBF were estimated using a mixed modeling procedure for analysis of variance to estimate changes in means of variables between groups (SAS Software System). MAP was analyzed using repeated measures two-way analysis of variance (RM ANOVA).

## Results

### Mean arterial pressure

**Figure 1:**
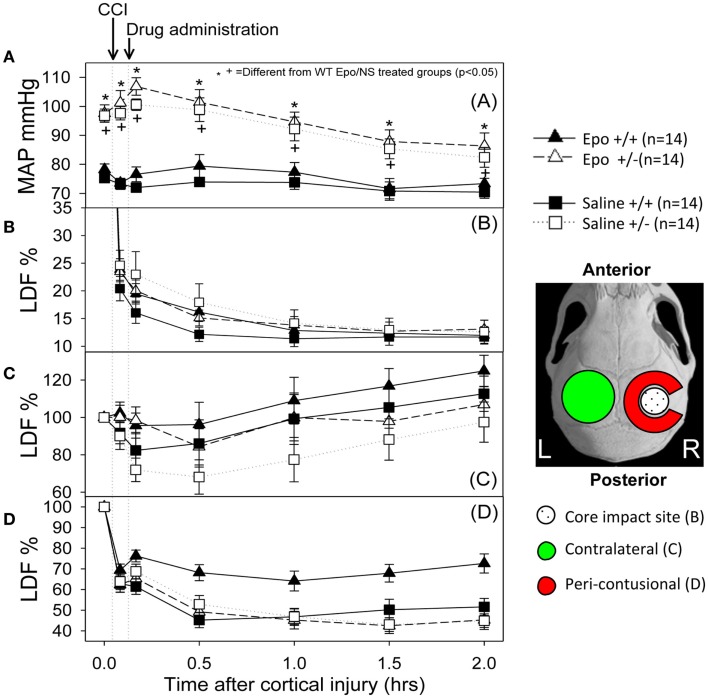
**Hemodynamic response to the controlled cortical impact (first arrow) followed by administration of the assigned treatment (second arrow)**. **(A)** Mean arterial pressure (MAP) changes during the experiment. The animals that were deficient in eNOS had a higher pre-injury MAP and had a greater drop in MAP after injury than the wild-type animals. When the group × time interaction was significant, symbols indicate which treatments were significantly different among the treatments’ groups by the Holm-Sidak *post hoc* test. **(B–D)** Cerebral blood flow response measured by laser Doppler in the core **(B)**, contra-lateral **(C)** and peri-contusional **(D)** cerebral cortex. The core had a severe persistent drop in CBF regardless of the animal genotype or treatment group. The contra-lateral cortex showed a small to no decline followed by increased perfusion in all groups. In the peri-contusional regions, Epo treatment effect was seen in the WT animals but not in the eNOS-deficient animals (*N* = 14 per group).

Figure 1 summarizes the changes in blood pressure that occurred following CCI. WT mice showed an initial MAP of 76.75 ± 1.4 mm Hg compared to 97.17 + 0.39 mm Hg in the eNOS-deficient animals (*p* < 0.05). eNOS-deficient mice started from a higher baseline blood pressure that remained consistently higher throughout the experiment because of tonically reduced vasodilatation, but these animals had a greater relative drop (15%) in MAP from baseline at the end of the 2 h monitoring period. Following treatment, WT animals had MAP measurements that remained closer to baseline values than the eNOS-deficient animals (genotype effect, *p* < 0.001; time effect, *p* < 0.001; time ? genotype interaction, *p* < 0.001). At the end of the monitoring period, MAP averaged 86.35 ± 4.48 and 82.42 ± 3.42 mm Hg in the Epo/Saline eNOS deficient treated groups, respectively; compared to 73.35 + 1.83 and 70.42 ± 2.09 mm Hg in the Epo/Saline WT treated animals, respectively (*p* < 0.05, Holm-Sidak test).

### Cerebral hemodynamics

Following cortical injury, CBF decreased in all treatment groups, with the greatest reductions occurring at the core impact site with less reduction in the peri-contusional cortex and least in the contra-lateral cortex. CBF fell in the impact site from 100% to a low value between 10 and 15% in all treatment groups by 2 h after cortical injury. No significant blood flow differences were found over time or by genotype at the core impact site (*p* > 0.05) (Figure 1B). Although not significant, a trend for better CBF recovery contra-lateral to the injury was observed over time favoring the WT animals (Figure 1C). Significant genotype and treatment differences in CBF were found only in the peri-contusional sites. Reduction in CBF values in the peri-contusional sites was similar between treatment groups immediately after cortical injury. Subsequently, peri-contusional CBF declined to 50% of baseline in the saline-treated animals and the Epo-treated eNOS-deficient animals, but Epo administration resulted in significantly higher CBF recovery in the peri-contusional region in the WT mice (70 ± 3.3% in Epo-treated groups compared to 53 ± 3.35% in saline-treated mice; *p* < 0.05) (Figure 1D). The results of the statistical analyses are summarized in Table 1. In the peri-contusional (penumbral) area, there is a significant treatment effect and genotype effect but the interaction indicates that each depends on the other. There is also a significant change over time that depends on the genotype. There is no effect of Epo in the eNOS ± genotype but the Epo effect is significantly greater in the eNOS WT genotype. These differences are most notable between pre-injury and pre-injection and the time points 30, 60, 90, and 120 min after injury.

**Table 1:** **Multi-level statistical model for the cerebral blood flow (CBF) data comparing the EPO-treated and the saline-treated groups in the two genotypes of mice**.

Effect	Core				Peri-contusional				Contra-lateral			
	NDF	DDF	F-value	*p*	NDF	DDF	F-value	p	NDF	DDF	F-value	*p*
Cerebral blood flow												
Treatment	1	52	0.7	0.4056	1	52	5.29	0.0255	1	52	2.77	0.1023
Genotype	1	52	0.92	0.3418	1	52	7.35	0.0091	1	52	2.13	0.1508
Treatment*genotype	1	52	0.65	0.4248	1	52	8.00	0.0066	1	52	0.08	0.7832
Time	5	260	80.59	<0.0001	5	260	39.26	<0.0001	5	260	10.22	<0.0001
Time*treatment	5	260	0.19	0.9652	5	260	1.09	0.3684	5	260	0.39	0.8548
Time*genotype	5	260	0.99	0.4221	5	260	6.91	<0.0001	5	260	1.32	0.2572
Time*treatment*genotype	5	260	1.50	0.189	5	260	1.11	0.357	5	260	0.35	0.8804

*NDF, numerator degrees of freedom; DDF, denominator degrees of freedom*.

*Multi-level statistical model of the CBF in the core, penumbra, and contra-lateral cerebral cortex. In the peri-contusional (penumbral) area, there is a significant treatment effect and genotype effect but the interaction indicates that each depends on the other. There is also a significant change over time that depends on the genotype. There is no effect of Epo in the eNOS ± genotype but the Epo effect is significantly greater in the eNOS WT genotype (*N* = 14 per group: eNOS ± treated with normal saline, eNOS ± treated with Epo, eNOS treated with normal saline, and eNOS treated with Epo)*.

## Discussion

Cerebral pressure autoregulation is the intrinsic ability to maintain constant CBF over a range of blood pressures, and metabolic cerebral autoregulation is the ability of the brain to locally adjust CBF to meet cerebral metabolic requirements (37). Cerebral pressure autoregulation is disrupted in TBI and this may be due to dysfunction of endothelial NO production.

Nitric oxide plays an important role in a number of general physiological processes of the brain, such as the maintenance of basal vasomotor tone, selective neuroprotection, synaptogenesis, and synaptic plasticity; therefore, NO has multiple and complex roles in the pathophysiology of TBI. NO is a cell membrane-permeable free radical synthesized from the amino acid l-arginine by the enzyme NOS (38). In TBI, both excess and deficiency of NO can potentially result in detrimental effects. Depletion of NO produced by eNOS may result in inadequate cerebral perfusion, whereas excess NO produced by nNOS and iNOS could potentially lead to neurotoxicity and cellular injury. Such changes in cerebral NO metabolism have been implicated in the pathophysiological changes occurring after TBI. Triphasic (high-low-high) changes in cerebral concentration of NO after TBI have been described in experimental and clinical studies. A brief rise in NO concentration lasting seconds to minutes immediately after injury in experimental models is followed by a protracted decrease (0.5–6 h) in NO concentrations that may partly account for the low CBF observed during this period after injury. This is followed by a late phase (>6 h) in which an elevation in cerebral NO is associated with a return to normal or even elevated levels of CBF. Increased expression of iNOS protein in cerebrovascular smooth muscles has been observed during this phase, and CSF levels of NO have been reported to peak between 20 and 42 h after TBI (39). During the early phase, a period of relative deficiency in NO and a low level of CBF, the administration of l-arginine has been shown to improve CBF and neurological outcome in models of TBI (34). Administration of l-arginine in patients after severe TBI induced an increase in internal carotid artery flow volume, which was larger at 48 h than at 12 h, and tended to be larger in the less injured hemisphere at both time periods, suggesting that dysfunction of cerebrovascular endothelium plays a role in the reduced CBF observed after TBI (40). Some of the variability in patient outcome that occurs following severe TBI may result from eNOS polymorphisms (41). During the late peak in NO after TBI due to the activity of iNOS, the inhibition of iNOS has been neuroprotective in experimental models of TBI (42–44). l-Arginine administration may or may not result in an increase of CBF after CCI depending upon whether or not the mice had been hemorrhaged (45). It is well documented that CBF and cerebral autoregulation are heterogeneous after TBI and tend to be reduced in the immediate vicinity of a contusion (46).

Our experimental model focuses on the early phase after CCI and previous studies have shown a lower plasma and microdialysate NO concentration in the eNOS-deficient mice compared to WT mice. Our study also showed a sustained decrease in post-traumatic CBF in eNOS-deficient mice compared to WT mice. eNOS-deficient mice had significant lower CBF in peri-contusional areas of the brain in spite of maintaining higher MAP compared to WT mice. Moreover, earlier studies have demonstrated that eNOS-deficient mice have higher MAP than do WT mice but comparable CBF (47), these finding have been confirmed in the present study. It has also been shown that cortical blood flow, measured by laser Doppler, is reduced by about 70% from baseline in eNOS-deficient mice and by about 50% in WT mice 2 h after trauma (48, 49). These values are comparable to the relative reduction in blood flow observed at 2 h after CCI in our present study. CBF changes detected by LDF were only consistently significant in the peri-contusional areas of the brain (e.g., penumbra), which are vulnerable to additional secondary insults (e.g., hypoxia, hypotension). Importantly, these same areas are often considered as potentially salvable cerebral tissue that could contribute in the recovery and outcome after TBI.

Discovery of Epo and EpoR in many non-erythroid organs and tissues such as endothelial cells, reproductive organs, heart, gastrointestinal tract, muscle cells, and the central nervous system (CNS) suggests a wide variety of actions (23, 50, 51). Different cell types (neurons, glia and endothelial cells) in the nervous system produce Epo and express EpoR, and Epo appears to have multiple actions in the nervous system. Epo has neuroprotective effects after ischemic, hypoxic, metabolic, neurotoxic, and excitotoxic stress in the nervous system. This neuroprotection potentially operates simultaneously at several levels in the central nervous system, such as limiting the production of reactive oxygen species and glutamate, neurotransmission modulation, reversal of vasospasm, promoting angiogenesis, preventing apoptosis, reduction of inflammation, and stem cell recruitment. Our study indicates that some of the neuroprotective effect results from maintenance of CBF by an eNOS dependent mechanism. Similarly, NOS dependency of the neuroprotective effects of Epo has been described in the restoration of working memory deficits in rats subjected to global transient ischemia (52). Interestingly, in the stable tubule only polypeptide (STOP) null mouse model that displays neurochemical and behavioral aspects of schizophrenia, Epo results in an improvement in cognitive function that is NO dependent. These studies of working memory restoration in transient global ischemia and cognitive function in STOP mice used pharmacological inhibition of NOS, so it is not possible to discern if the effects were mediated by eNOS or nNOS.

Studies of the effects of Epo in organs other than the brain as well as *in vitro* endothelial cells suggest that protective effects are eNOS dependent. In cultured lung endothelial cells, hypoxia leads to constriction but in the presence of Epo there is up-regulation of eNOS with increased NO production leading to vasorelaxation. In addition, the NO leads to up-regulation of EpoR in these cells suggesting a coordinated response of these signaling molecules (53). In studies of sepsis in which tumor necrosis factor induces microvascular inflammation in striated muscle, Epo ameliorates the microvascular damage by an eNOS dependent mechanism but the protective effect is independent of iNOS (54). Similarly, Epo prevents sepsis-related acute kidney in rats by up-regulating eNOS (55). In ischemic musculocutaneous skin flaps, Epo administration up-regulates eNOS and the protective effect is abrogated by administration of eNOS inhibitors (56). Administration of Epo has also been shown to reduce the depth and area of secondary burn progression (57). These extra-hematopoietic eNOS dependent protective actions may result, at least in part, from vasodilation with increased blood flow.

The purpose of the present study is to identify the effect of Epo on CBF changes measured by LDF after TBI and the dependence of these changes on eNOS. Binding of Epo to its receptor (EpoR) on the endothelial cells of the cerebral vasculature leads to phosphorylation of JAK-2 that activates the PI3-K/AKT pathway resulting in eNOS activation by phosphorylation. The increased activity of eNOS increases NO production resulting in cerebral vasodilation and increased CBF. Epo administration resulted in significantly higher LDF in the peri-contusional sites only in the WT mice and eNOS-deficient mice showed poorer LDF recovery after injury in the peri-contusional sites with both treatment groups (Epo vs. saline; *p* > 0.05) having comparable LDF values at 120 min after CCI. The relatively rapid increase in CBF following Epo administration after CCI is likely due to direct signaling pathways in the cerebral vasculature leading to eNOS activation and NO production rather than other slower Epo effects that depend on transcriptional regulation. The rapid time course of changes in CBF also indicates direct physiological effect rather than impaired vascular remodeling in the eNOS-deficient animals or up-regulation of inducible or neuronal NOS. Previous studies have shown that restoration of penumbral blood flow using NO augmentation is associated with better behavioral, histological, and contusion volume outcome following CCI. A recent study using inhaled NO in the CCI suggests that CBF can be restored without disrupting cerebral autoregulation, increasing intracranial pressure, or protein nitrosylation, and that augmenting NO by this means results in reduced contusion volume and better behavioral outcome (58).

There are some technical limitations of this study. CBF was measured using laser Doppler which provides arbitrary PUs rather than quantitative CBF that can be expressed in ml/mg/min; therefore, we measured relative perfusion. The major advantage of using laser Doppler is that this technique enables rapid serial measurements. Lundband et al. measured cortical blood flow following CCI in eNOS-deficient mice using [14*C*]-iodoantipyrine at 3 and 24 h after injury (49). The magnitude of CBF change they observed at 3 h was similar to that we observed at 30 min, 1 h and 2 h after injury.

Our study supports the importance of eNOS and NO in the very early stages in the pathophysiology of TBI in maintaining adequate CBF as well as the positive effect of Epo in re-establishing the CBF in the penumbra within 2 h after the injury by a NO dependent mechanism. These findings support the potential for very early administration of Epo or Epo agonists in the management of TBI.

## Conflict of Interest Statement

The authors declare that the research was conducted in the absence of any commercial or financial relationships that could be construed as a potential conflict of interest.
